# Serum ferritin as a predictive marker of pulmonary fibrosis in post-COVID-19

**DOI:** 10.5339/qmj.2025.44

**Published:** 2025-06-09

**Authors:** Aditya Ojha, Muskan Bhasin, Megha Bhat Agni, KM Damodara Gowda

**Affiliations:** 1 K. S. Hegde Medical Academy, Nitte (Deemed to be University), Deralakatte, Mangalore, India; 2 Department of Physiology, K. S. Hegde Medical Academy, Nitte (Deemed to be University), Deralakatte, Mangalore, India; 3 Department of Physiology, Siddaganga Medical College & Research Institute, Tumakuru, Karnataka-572102, India *Email: damodar001@gmail.com

**Keywords:** COVID-19, CT severity score, Ferritin, Plateletcrit, Platelet Distribution Width, Red cell Distribution Width

## Abstract

**Background:**

Pulmonary fibrosis is characterized by excessive matrix formation, which destroys typical lung architecture and increases the chances of comorbidity. It is essential to look into potential serum indicators for the early identification of individuals who may develop such severe fibrotic consequences since there is currently no specific marker for the early diagnosis of post-COVID-19 pulmonary fibrosis. The study is aimed at examining potential serum markers that could be used for early detection of pulmonary fibrosis in patients with COVID-19.

**Methods:**

It is a cross-sectional retrospective observational study that included male (*n* = 26) and female (*n* = 10) patients who were confirmed positive for COVID-19 using the Reverse transcription polymerase chain reaction (RTPCR) test. Various hematological parameters, such as platelet count, white blood cell count (WBC count), platelet-to-lymphocyte ratio (PLR), white blood cell count to mean platelet volume ratio (WMR), red cell distribution width (RDW), plateletcrit (PCT), mean platelet volume (MPV), platelet distribution width (PDW), serum ferritin level, and CT severity scores (CT-SSs) were recorded. The association between hematological parameters, serum ferritin level, and CT-SS was assessed by the Pearson correlation test using the GraphPad Prism software (version 10). *p* < 0.05 was considered statistically significant.

**Results:**

The descriptive analysis revealed no significant correlation between platelet count (*r* = 0.1610, *p* = 0.3483), WBC count (*r* = −0.1381, *p* = 0.4217), PLR (*r* = 0.2262, *p* = 0.1847), WMR (*r* = −0.1093, *p* = 0.5258), RDW (*r* = 0.05982, *p* = 0.7289), PCT (*r* = −0.059, *p* = 0.752), MPV (*r* = 0.046, *p* = 0.788), and PDW (*r* = −0.06, *p* = 0.699) with CT-SS. However, a significant positive correlation was observed between CT-SS and serum ferritin levels in COVID-19 patients (*r* = 0.5452, *p* = 0.0006).

**Conclusions:**

As there was a significant positive correlation between serum ferritin level and CT-SS, the serum ferritin level could be considered as a simple and cost-effective biomarker for predicting the development of lung fibrosis in long COVID-19 conditions after controlling the confounders.

## Introduction

The World Health Organization has reported 700 million cases of COVID-19 worldwide and 6.96 million deaths by the end of 2023 since the start of the pandemic.^1^ Although most people fully recover from SARS-CoV-2 infection, a subset of COVID-19 patients have unexplained persistent residual symptoms or new symptoms. This disease is known as long COVID or post-COVID-19 condition or acute consequences of SARS-CoV-2 infection. The consequences of COVID-19 may vary from minor fatigue to severe conditions necessitating long-term oxygen treatment or maybe a lung transplant owing to pulmonary fibrosis.^2^

Common instances of COVID-19 infection were characterized by people exhibiting fever, respiratory symptoms, and pneumonia as evident by imaging. In critical instances, one of the following three clinical symptoms is evident: (a) dyspnea accompanied by a respiratory rate exceeding 30 breaths per minute, (b) mean oxygen saturation at rest <9 3%, and (c) arterial oxygen partial pressure ≤30 mmHg.^3^

In the search for early predictive signs of pulmonary fibrosis, various parameters were evaluated in post-COVID-19 patients, and radiological imaging was correlated to assess the extent of fibrosis in patients. Depending on the severity of the initial infection, different study designs have been performed to identify these markers to limit the progression of fibrosis.^4^ An investigation conducted in Wuhan explored a retrospective examination of blood samples from 485 patients to ascertain mortality risk factors.^5^ Utilizing mathematical modeling, the authors found three indications (LDH, hs-CRP, and lymphocytes) for predicting COVID-19. Certain investigations have shown the presence of fibrogenesis biomarkers in the bronchoalveolar fluid 24 hours post-ARDS start, which is associated with mortality rates. This encompasses the N-terminal propeptide of collagen type III, the C-terminal propeptide of collagen type I, TGF-β, as well as alveolar fibroblasts and fibrocytes.^6^

Platelets are crucial in thrombosis since they are swiftly activated and aggregate at locations of vascular damage. Transient platelet activation elicits proinflammatory and profibrotic responses via the release of powerful vasoactive neurotransmitters, inflammatory cytokines, and profibrotic substances from dense and α-granules.^7^ Enhanced platelet activation has been seen in adult respiratory distress syndrome (ARDS) and chronic obstructive pulmonary disease (COPD).^8^ Activated platelets forfeit their capacity to preserve the integrity of vessel walls, hence increasing the permeability of the pulmonary microvasculature. Elevated levels of platelet activation markers are seen in both mild and severe instances over both short and extended durations. Consequently, the risk of thrombotic events may persist indefinitely. Injury to the vascular endothelium, immune cells, inflammatory mediators, and hypoxia contribute to elevated platelet reactivity and aggregation via various mechanisms.^9^ Neutrophils and platelets, constituents of the immune system identified in the complete blood count, are pivotal in host defense. Activated cells induce the production of cytokines, resulting in the activation of both these cells and other cells of the immune system. The function of platelets in idiopathic pulmonary fibrosis (IPF) remains unclear. However, fibrin production and degradation are disrupted in the lungs of individuals with IPF. Excessive local fibrin deposition, which is crucial for fibroblast adhesion and proliferation, may lead to pulmonary fibrosis.^10^ Parameters associated with platelet size indicated platelet activity and were termed platelet indices, which include mean platelet volume (MPV), platelet distribution width (PDW), and plateletcrit (PCT).^11^

The production of ferritin is intricately influenced by various oxidant and antioxidant factors, such as nitrous oxide, glutathione, and reactive oxygen species. As an acute-phase protein, serum ferritin acts as a key marker for both chronic and acute inflammatory responses in the body.^12^ However, the link between hyperferritinemia and inflammation remains unclear.^13^ Increased ferritin levels point to an activated monocyte-macrophage system, suggesting the presence of inflammation. In monocytes and macrophages, ferritin synthesis is closely regulated by cytokine levels through transcriptional and translational mechanisms. Despite this understanding, the exact process by which intracellular ferritin transitions to serum ferritin is not yet fully understood. Pretorius E & Kell DB, and Edeas et al. proposed that hyperferritinemia might result from the leakage of ferritin caused by damage to intracellular stores.^14,15^ When ferritin is released from tissues, it often lacks its iron core, leading to an accumulation of free iron in the body. This surplus of iron can not only promote fibrin polymerization but also contribute to a pro-coagulant state.^16^ Gaining deeper insights into this complex relationship is crucial for mitigating the health risks associated with elevated ferritin levels.

Iron is crucial for several metabolic activities and is toxic in excess due to its ability to catalyze the production of reactive oxygen species, which can cause oxidative stress and damage cell membranes.^17^ Ferritin, as an acute-phase reactant, can be identified in many acute and chronic inflammatory conditions, perhaps resulting from the release of compromised intracellular reserves. Upon the release of ferritin, it decomposes and liberates surplus free iron, hence facilitating the proliferation and reproduction of several viruses. Numerous regulatory and functional proteins of the SARS-CoV-2 virus use iron. Increased serum ferritin levels may reflect abnormal production of ferritin due to macrophage activation, leading to fiber accumulation in the lung parenchyma.^18^

As SARS-COV-2 infection leads to an inflammatory process and immune activation, blood parameters can be used to correlate CT severity scores (CT-SSs) in post-COVID-19 patients to identify prognostic biomarkers. Although serum indicators such as WBC count, platelet-to-lymphocyte ratio (PLR), white blood cell count to mean platelet volume ratio (WMR), red cell distribution width (RDW), PCT, serum ferritin level were often used for the early identification of individuals who may develop severe fibrotic consequences, the correlation of these parameters with CT-SS is lacking. Therefore, we hypothesize that platelet parameters and serum ferritin levels in patients with COVID-19 are positively correlated with CT severity. Therefore, the aim of this study was to investigate the early predictors of pulmonary fibrosis by examining the relationship between platelet parameters, including MPV, PDW, PCT, PLR, WMR, RDW, and serum ferritin level with the severity of lung injury as assessed by the CT-SS.

## Subjects and Methods

### Sample and setting

The present cross-sectional retrospective study included 36 patients confirmed by RT-PCR test without prior vaccination for COVID-19. The current study used RECORD guideline— “REporting of studies conducted using Observational Routinely-collected Data” from the medical records department of our tertiary care hospital. A total of 36 patients with an age range from 19 to 75 years old were selected. The clinical data of all patients who attended our tertiary care hospital was procured. Patients with positive COVID-19 CT chest findings and whose results were verified by a PCR test were included. Patients with severe respiratory motion artifacts on CT images, patients with a history of chronic interstitial lung disease, and patients with other chronic medical conditions such as diabetes mellitus, hypertension, and autoimmune disease were excluded. Incomplete medical record data were also excluded from the study. The study was approved by the institutional review board bearing the number EC/125/2021-22 before the data collection and was conducted in accordance with the principles set forth in the Helsinki Declaration.

### Data collection

The data was obtained at the medical records department from the patients who had been admitted and had undergone non-contrast-enhanced chest CT in our tertiary care hospital between April 2020 and June 2021. Various CT results, including ground glass opacities, vascular thickening, and bronchial thickening, were evaluated in terms of their location and distribution. CT imaging characteristics, like bronchovascular pack deformation, fibrotic stripes, traction bronchiectasis, architectural distortion, and interlobar septal thickening, indicate pulmonary fibrosis. CT images were analyzed by expert thoracic imaging radiologists, and the CT scores were awarded. Patients in the current study were categorized on the basis of CT-SS as mild, moderate, and severe, each depicting the extent of the fibrosis in each of the categories. All the data collected were used in the study for analysis and were represented as figures and tables.

### CT severity scores (CT-SS)

This score assesses the extent of lung involvement by partitioning the lung into five lobes. Each lobe’s involvement is visually rated on a scale from 0 to 5, where 0 signifies no involvement, 1 denotes less than 5% involvement, 2 indicates 5–25% involvement, 3 represents 26–49% involvement, 4 corresponds to 50–75% involvement, and 5 reflects more than 75% involvement. The cumulative CT score was the aggregate of the individual lobar scores, ranging from 0 (no participation) to 25 (maximum involvement). The CT-SS was defined as the sum of the individual scores in the 20 lung segment regions, ranging from 0 to 40 points, which is categorized as mild (<8), moderate (9–15), and severe (>15).

### Hematological parameters

The platelet parameters, including the MPV, PDW, PCT, PLR, WMR, RDW, and serum ferritin levels, were collected from the selected patients from the medical records department. The hematological parameters were measured using the Beckman Coulter electrical impedance method. Serum ferritin level was measured by radioimmunoassay. The data were documented as Mean and Standard deviation.

### Statistical analysis

The data was represented as medians and interquartile ranges owing to the limited sample size, whereas categorical variables are represented as whole numbers accompanied by percentages in brackets. Quantitative variables were documented as mean, standard deviation, and confidence interval. The association between the hematological and serum ferritin levels with CT-SS was assessed using the Pearson correlation. *p* < 0.05 was considered statistically significant.

## Results

**Figure 1:**
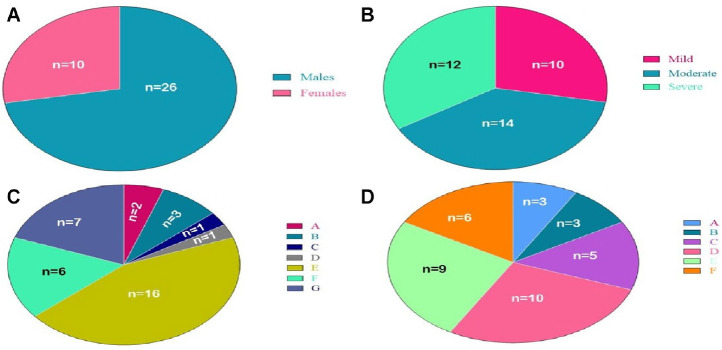
Demographic features of recruited patients. (A) indicates the gender-wise distribution of patients. (B) indicates the number of patients based on the severity of COVID-19. (C) indicates the number of patients based on the past history of comorbidities. (D) indicates the number of patients based on the age-wise distribution of patients.

The present study included thirty-six patients, ten women and twenty-six men (Figure 1A) and was divided into three categories based on CT-SSs: mild (*n* = 10), moderate (*n* = 14), and severe (*n* = 12) patients (Figure 1B). Figure 1C shows the past history of enrolled patients, where “A” indicates a known case of bronchial asthma (*n* = 2), “B” indicates a known case of diabetes mellitus with systemic hypertension (*n* = 3), “C” indicates a known case of diabetes mellitus, systemic hypertension, and pulmonary tuberculosis (*n* = 1), “D” indicates known systemic hypertension and coronary disease (*n* = 1), “E” denotes patients without known comorbidities (*n* = 16), “F” shows a known case of hypertension (*n* = 6), and “G” represents a known case of type 2 diabetes (*n* = 7). The age distribution of patents is shown in Figure 1D, where “A” indicates that patients belong to the age group of 15–25 years (*n* = 30), “B” indicates that patients belong to the age group of 26–35 years (*n* = 3), “C” indicates that patients belong to the age group of 36–45 years (*n* = 5), “D” indicates that patients belong to the age group of 46–55 years (*n* = 10), “E” indicates that the patients belong to the age group of 56–65 years (*n* = 9) and “F” indicates that the patients belong to the age group of 66–75 years (*n* = 6).

The platelet parameters, including MPV, PDW, PCT, PLR, WMR, RDW, and serum ferritin levels, were thoroughly examined in relation to the CT-SS (CO-RADS). The comprehensive findings are presented in Table 1, providing an overview of the observed correlations. It is worth noting that the correlation between the CT-SS and the various hematological parameters of COVID-19 patients did not exhibit any statistically significant differences, except for the serum ferritin levels, which indicated a significant positive correlation (*p* = 0.0006), as highlighted in Table 1. This finding suggests a potential association between the severity of CT scans and the levels of serum ferritin in COVID-19 patients. The correlation of mild CT-SS with hematological parameters of COVID-19 patients did not show a significant correlation, whereas the platelet count showed a significant positive correlation (Table 2). The correlation of moderate (Table 3) and severe (Table 4) CT-SS with hematological parameters of COVID-19 patients did not show a significant correlation.

**Figure 2:**
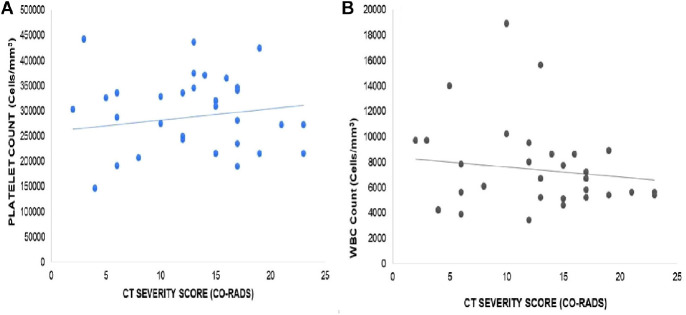
Correlation of CT-SS (CO-RADS) with platelet count in COVID-19 patients, *r* = 0.1610, *p* = 0.3483 (A), and WBC count, *r* = −0.1381, *p* = 0.4217 (B). The correlation is insignificant.

**Figure 3:**
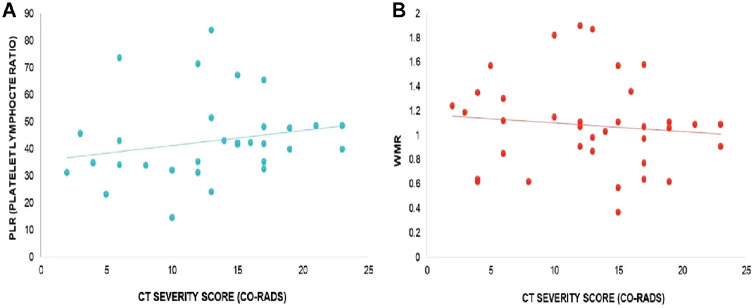
Correlation of CT-SS (CO-RADS) with PLR in COVID-19 patients, *r* = 0.2262, *p* = 0.1847 (A), and WMR, *r* = −0.1093, *p* = 0.5258 (B). The correlation is insignificant.

**Figure 4:**
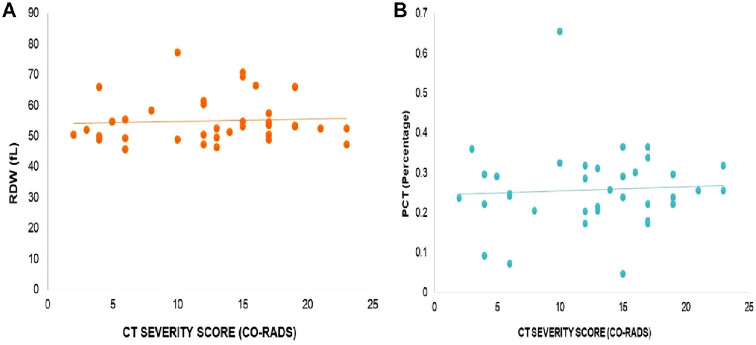
Correlation of CT-SS (CO-RADS) with RDW in COVID-19 patients, *r* = 0.05982, *p* = 0.7289 (A), and PCT, *r* = −0.059, *p* = 0.752 (B). The correlation is insignificant.

**Figure 5:**
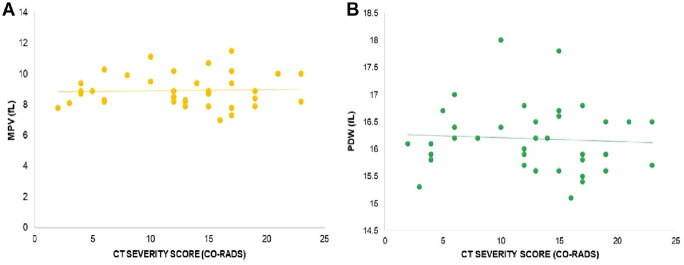
Correlation of CT-SS (CO-RADS) with MPV in COVID-19 patients, *r* = 0.046, *p* = 0.788 (A), and PDW, *r* = −0.06, *p* = 0.699 (B). The correlation is insignificant.

Furthermore, the correlation between the CT-SS (CO-RADS) and platelet count was found to be statistically insignificant (*r* = 0.1610, *p* = 0.3483, as illustrated in Figure 2A). Similarly, no significant correlations were observed between the CT-SS and other hematological parameters, including WBC count (*r* = −0.1381, *p* = 0.4217, Figure 2B), PLR (*r* = 0.2262, *p* = 0.1847, Figure 3A), WMR (*r* = −0.1093, *p* = 0.5258, Figure 3B), RDW (*r* = 0.05982, *p* = 0.7289, Figure 4A), PCT (*r* = −0.059, *p* = 0.752, Figure 4B), MPV (*r* = 0.046, *p* = 0.788, Figure 5A), and PDW (*r* = −0.06, *p* = 0.699, Figure 5B). These findings indicate that there is no significant relationship between the CT-SS and the aforementioned hematological parameters in COVID-19 patients, as the correlations were not statistically significant.

**Figure 6:**
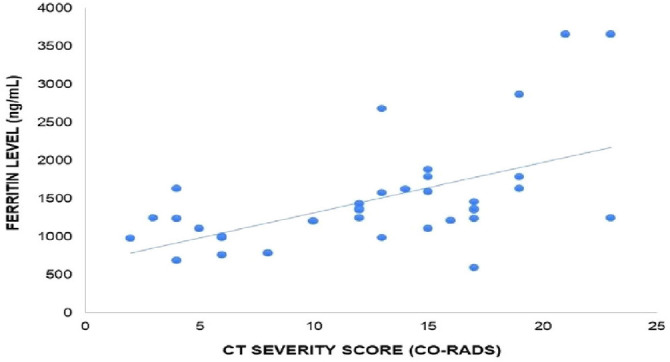
Correlation of CT-SS (CO-RADS) with serum ferritin level in COVID-19 patients, *r* = 0.5452, *p* = 0.0006. There is a significant positive correlation between ferritin level and CT severity score.

**Figure 7:**
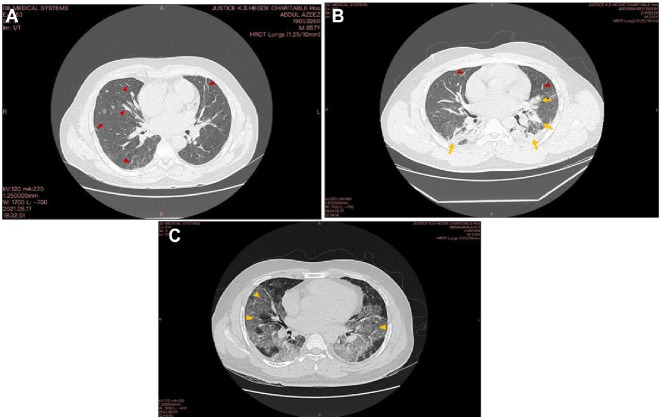
Representative images of axial high-resolution chest CT of the mild, moderate, and severe conditions of a diagnosed COVID-19 patient with CO-RAD 6 (A) indicates the axial HRCT showing bilateral fibrotic bands (red arrowheads) with predominantly peripheral distribution with a CT-SS of 6/25 (mild). (B) indicates the axial HRCT showing consolidations in both the lung fields (yellow arrows) and fibrotic bands (red arrowheads) with predominantly peripheral distribution with a CT-SS of 15/25 (moderate). (C) indicates axial HRCT showing bilateral patchy ground glass opacities (yellow arrowheads) with predominantly peripheral and peribronchovascular distribution with a CT-SS of 21/25 (severe).

The correlation between the CT-SS (CO-RADS) and the serum ferritin level in COVID-19 patients exhibited a significant positive correlation (*r* = 0.5452, *p* = 0.0006, Figure 6). This finding suggests that higher levels of serum ferritin may be an indicator of more severe CT scans in COVID-19 patients. Further research is warranted to explore the underlying mechanisms and potential clinical implications of this correlation. The representative images of axial high-resolution chest CT of mild, moderate, and severe conditions of a diagnosed COVID-19 patient with CO-RAD-6 (Figure 7). The patients with mild CT-SS showed bilateral fibrotic bands with predominantly peripheral distribution (Figure 7A), patients with moderate CT-SS showed consolidations in both the lung fields and fibrotic bands with predominantly peripheral distribution (Figure 7B), and patients with severe CT-SS showed bilateral patchy ground glass opacities with predominantly peripheral and peribronchovascular distribution (Figure 7C). The results showed a significant positive correlation with the platelet count in patients having mild CT-SS.

## Discussion

The present study showed a significant positive correlation with serum ferritin levels and CT-SSs. The ferritin level ranges between 24–336 ng/mL in males and 24–307 ng/mL in females, and we found the mean ferritin level as 1486 ± 707.18 ng/mL. Enomoto N et al. reported that the patients with much higher ferritin (≥500 ng/mL) had significantly worse prognoses than those with lower ferritin.^19^ Platelet markers showed a non-significant correlation with CT severity. Our results are consistent with those of Chong DWL et al.,^20^ who reported that pulmonary fibrosis does not pose a risk even when the platelet count is higher than normal. They found that IPF patients had more platelets, neutrophils, and active TGF-β in the airways than controls. These authors used an animal model of IPF to show that platelet-specific TGF-β does not induce lung inflammation or fibrosis and found no significant association between CT-SSs and any parameter describing platelet count.

The retrospective data from the medical records section showed that patients with mild CT-SS did not require ventilator support and were managed with oxygen support, whereas patients with moderate and severe CT-SSs were intubated and with prolonged ventilatory support during the hospital stay. The retrospective data also revealed characteristic COVID-19-induced pathological changes in the lungs, which were regarded as the cause of death in most patients. The main histological finding reported was sequential alveolar damage, leading to the death of the patient either directly or by the induction of pulmonary parenchymal fibrosis. Diffuse lung damage was seen exclusively in invasively ventilated patients.^21^

The prevalence of fibrosis after COVID-19 is emerging gradually, but according to previous studies of patients discharged from hospitals after COVID-19, almost one-third of recovered patients developed an abnormality related to fibrosis.^2^ Thille et al. showed a 4% incidence of fibrosis in ARDS patients who died within 1 week, 24% of cases where death occurred between 1 and 3 weeks, while those who died more than three weeks had the highest impact (61%) of fibrosis.^22^

Platelet parameters, including MPV, PDW, PCT, PLR, WBC: MPV ratio, and RDW, were not correlated with CT severity in this study. According to Makhlouf HA et al., high values of these indices are associated with higher levels of inflammation in the body and severity and acute exacerbation of COPD.^23^ The MPV indicates the presence of young platelets in the blood since the larger ones have a relatively higher metabolic activity than the smaller ones and are more active. In addition, MPV has been found to be an indicator of cardiovascular occlusion and inflammatory disease.^24^ Higher PDW values are associated with larger platelet size, which may be due to mechanisms of platelet destruction, activation, or consumption processes.^25^ Cretin levels in platelets increased during inflammation. We found no significant correlation between CT-SSs and RDW, a numerical measure of variability in circulating erythrocyte size. RDW can increase after systemic inflammatory diseases, ineffective erythropoiesis, nutritional deficiencies, bone marrow dysfunction, or even increased destruction of red blood cells, which can lead to higher RDW in healthy individuals.^26^ It has been reported that RDW increases with the progression of COPD severity, and it has been suggested that RDW could be used as a biomarker to monitor the severity of the disease.^26^ PLR and neutrophil-to-lymphocyte ratio have also been found to be associated with COPD progression and severity.^27^

In patients with COVID-19, platelets are highly activated, which can damage blood vessels and lead to the formation of fibrous tissue. As shown in our study, we found no significant correlation between CT-SSs and platelet counts. Chong DWL et al. also found similar findings suggesting that the risk of pulmonary fibrosis does not increase even with increased platelet count,^17^ while Michael G. et al. reported that indirectly activated platelets release fibrogenic factors such as PDGF and TGF-α, which caused fibrous tissue deposition, but did not confirm the association between platelet activation and the occurrence of pulmonary fibrosis.^28^ A negative correlation with platelet count, critical platelet level, MPV, and PDW was reported in patients who subsequently developed pulmonary fibrosis. MPV showed higher levels in non-COVID-19 patients and those younger than 65 years.^29^

However, our study showed that in patients over 60 years of age, MPV is positively correlated with pulmonary fibrosis, in contrast to our findings of PDW, which neither increased nor decreased with pulmonary fibrosis.^30^ In this regard, ferritin is very important in assessing the severity and response of COVID-19 pneumonia during hospitalization. COVID-19 lungs were stabilized by changing ferritin titer during hospitalization and at discharge; Thus, early pulmonary fibrosis after COVID-19 can be predicted.^31^

There was no significant difference in CT-SSs (CO-RADS) in patients with COVID-19 with different platelet parameters and serum ferritin levels, except for serum ferritin levels. However, it is worth noting that there was no significant correlation between CT-SSs and other hematological parameters, which means that they may not be good indicators of the level of CT scans in COVID patients.

## Conclusion

We demonstrated a significant positive correlation between serum ferritin levels and CT severity, a commonly used measure of pulmonary fibrosis severity. Our study emphasizes the importance of measuring serum ferritin levels to obtain important vital about the further progression of pulmonary fibrosis, which helps to develop a successful treatment strategy. Such an assessment could be used as an early indicator of pulmonary fibrosis after COVID, taking into consideration of confounding factors, allowing doctors to take early steps to stop or slow the progression of this crippling disease. However, it is important to acknowledge that the sample size of this study was small, which limits the broader applicability of the current findings. Thus, more studies, including a larger and more diverse sample, are warranted to demonstrate a broader application of our results.

## List of abbreviations

MPV Mean platelet volume

PCT Plateletcrit

PDW Platelet distribution width

PLR Platelet to lymphocyte ratio

RDW Red cell distribution width

WMR White blood cell count to mean platelet volume ratio

## Conflicts of interest

The authors certify that there is no conflict of interest with any financial organization regarding the material discussed in the manuscript.

## Funding

This research was funded by a grant from Nitte (Deemed to be University) (N/RG/NUSR2/KSHEMA/2021/03) to the first author as Nitte University Student Research grant.

## Figures and Tables

**Table 1.** Correlation of CT-SS with hematological and serum ferritin levels in patients diagnosed with COVID-19.

	**Variables with normal range**	**Mean ± SD**	**95% confidence interval**		**Pearson correlation**		**2-tailed *p*-value**	**Significance**
			**Lower**	**Upper**	**R value**	**R squared**		
**Overall correlation of CT-SS with**	Platelet count (1.5–4.0 lakhs cells/mm^3^)	287875 ± 82351.28	−0.1769	0.4649	0.1610	0.02592	0.3483	NS
	WBC count (4000–11000 cells/mm)	7388.88 ± 3315.65	−0.4464	0.1994	−0.1381	0.01908	0.4217	NS
	PLR 117.05 ± 47.73	42.69 ± 14.18	−0.1106	0.5163	0.2262	0.05115	0.1847	NS
	WMR 760 ± 21	1086 ± 37	−0.4226	0.2274	−0.1093	0.01194	0.5258	NS
	RDW (12.2–16.1%)	54.99 ± 7.57	−0.2741	0.3809	0.05982	0.00358	0.7289	NS
	PCT (0.22–0.24 %)	0.257 ± 0.10	−0.2746	0.3804	0.059	0.00351	0.732	NS
	MPV (7.2 and 11.7 fL)	8.93 ± 1.07	−0.2866	0.3692	0.046	0.0021	0.788	NS
	PDW (8–12 fL)	16.19 ± 0.63	−0.3886	0.2679	−0.066	0.0044	0.699	NS
	Ferritin (24–336 ng/mL for males, and 24–307 ng/mL for females)	1486 ± 707.18	0.2639	0.7410	0.5452	0.2972	0.0006	***

Note: The overall correlation of CT-SS with hematological parameters of COVID-19 patients did not show a significant correlation except with that of serum ferritin levels, which showed a significant positive correlation. ^***^: highly significant, NS: non-significant.

**Table 2.** Correlation of mild CT-SS with hematological, and serum ferritin levels in patients diagnosed with COVID-19.

	**Variables**	**95% confidence interval**		**Pearson correlation**		**2-tailed *p*-value**	**Significance**
		**Lower**	**Upper**	**R value**	**R squared**		
**Correlation of mild CT-SS with**	Platelet count (cells/mm^3^)	0.1973	0.9737	0.8274	0.6846	0.0216	*
	WBC count (cells/mm^3^)	−0.124	0.9503	0.6939	0.4815	0.0838	NS
	PLR	−0.4809	0.8928	0.4267	0.182	0.3398	NS
	WMR	−0.4216	0.907	0.4857	0.2359	0.2692	NS
	RDW (%)	−0.7102	0.7904	0.3008	0.09045	0.5122	NS
	PCT (%)	−0.5223	0.881	0.3803	0.1447	0.4	NS
	MPV (fL)	−0.7102	0.7904	0.09213	0.00848	0.8443	NS
	PDW (fL)	−0.8626	0.576	−0.3127	0.0978	0.4947	NS
	Ferritin (ng/mL)	−0.7307	0.7738	0.04692	0.0024	0.9159	NS

Note: The correlation between mild CT-SS and hematological parameters of COVID-19 patients did not show a significant correlation except with platelet count, which showed a significant positive correlation. *: indicates significant at *p* < 0.05 level.

**Table 3.** Correlation of moderate CT-SS with hematological, and serum ferritin levels in patients diagnosed with COVID-19.

	**Variables**	**95% confidence interval**		**Pearson correlation**		**2-tailed *p*-value**	**Significance**
		Lower	Upper	R value	R squared		
**Correlation of moderate CT-SS with**	Platelet count (cells/mm^3^)	−0.7464	0.2138	−0.357	0.1277	0.2098	NS
	WBC count (cells/mm^3^)	−0.8198	0.02569	−0.5119	0.262	0.0613	NS
	PLR	−0.1507	0.7739	−0.5119	0.263	0.0613	NS
	WMR	−0.7557	0.1932	−0.3759	0.1413	0.1413	NS
	RDW (%)	−0.3033	0.7007	0.2708	0.07335	0.349	NS
	PCT (%)	−0.6239	0.4224	−0.1395	0.01945	0.6344	NS
	MPV (fL)	−0.3075	0.6984	0.2666	0.07107	0.3569	NS
	PDW (fL)	−0.1022	0.793	0.4529	0.2052	0.1039	NS
	Ferritin (ng/mL)	−0.1993	0.753	0.3704	0.1372	0.1923	NS

Note: The correlation of moderate CT-SS with hematological parameters of COVID-19 patients did not show a significant correlation. NS: non-significant.

**Table 4.** Correlation of severe CT-SS with hematological, and serum ferritin levels in patients diagnosed with COVID-19.

	**Variables**	**95% confidence interval**		**Pearson correlation**		**2-tailed *p*-value**	**Significance**
		**Lower**	**Upper**	**R value**	**R squared**		
**Correlation of severe CT-SS with**	Platelet count (cells/mm^3^)	−0.7332	0.4975	−0.1924	0.03703	0.5943	NS
	WBC count (cells/mm^3^)	−0.6083	0.65	0.03457	0.001195	0.9245	NS
	PLR	−0.7751	0.4207	−0.02842	0.08076	0.4262	NS
	WMR	−0.4105	0.78	0.2955	0.08732	0.4071	NS
	RDW (%)	−0.4775	0.7451	0.2175	0.04731	0.5461	NS
	PCT (%)	−0.5164	0.7212	0.1677	0.02813	0.6433	NS
	MPV (fL)	−0.7337	0.4967	−0.1934	0.03738	0.5925	NS
	PDW (fL)	−0.8372	0.2636	−0.4389	0.1926	0.2045	NS
	Ferritin (ng/mL)	−0.4596	0.7552	0.2393	0.05727	0.5055	NS

Note: The correlation of severe CT-SS with hematological parameters of COVID-19 patients did not show a significant correlation. NS: non-significant.
